# Effect of professional dental prophylaxis on the surface gloss and roughness of CAD/CAM restorative materials

**DOI:** 10.4317/jced.53826

**Published:** 2017-06-01

**Authors:** Toshiko Sugiyama, Atsushi Kameyama, Tomoka Enokuchi, Akiko Haruyama, Aoi Chiba, Setsuko Sugiyama, Makoto Hosaka, Toshiyuki Takahashi

**Affiliations:** 1 Senior Assistant Professor, Division of General Dentistry, Tokyo Dental College Chiba Hospital, 1-2-2 Masago, Mihama-ku, Chiba, Japan; 2 Associate Professor, Department of Operative Dentistry, Cariology and Pulp Biology, Tokyo Dental College, 1-2-2 Masago, Mihama-ku, Chiba, Japan; 3 Student, Tokyo Dental College School of Dental Hygiene, 1-2-2 Masago, Mihama-ku, Chiba, Japan; 4 Senior Assistant Professor, Department of Operative Dentistry, Cariology and Pulp Biology, Tokyo Dental College, 2-9-18 Misaki-cho, Chiyoda-ku, Tokyo, Japan; 5 Assistant Professor, Division of General Dentistry, Tokyo Dental College Chiba Hospital, 1-2-2 Masago, Mihama-ku, Chiba, Japan; 6 Clinical Professor, Division of General Dentistry, Tokyo Dental College Chiba Hospital, 1-2-2 Masago, Mihama-ku, Chiba Japan; 7 Associate Professor and Head, Division of General Dentistry, Tokyo Dental College Chiba Hospital, 1-2-2 Masago, Mihama-ku, Chiba, Japan

## Abstract

**Background:**

This study aimed to evaluate the effect of dental prophylaxis on the surface gloss and roughness of different indirect restorative materials for computer-aided design/computer-aided manufacturing (CAD/CAM): two types of CAD/CAM composite resin blocks (Shofu Block HC and Estelite Block) and two types of CAD/CAM ceramic blocks (IPS Empress CAD and Celtra DUO).

**Material and Methods:**

After polishing the CAD/CAM blocks and applying prophylaxis pastes, professional dental prophylaxis was performed using four different experimental protocols (n = 5 each): mechanical cleaning with Merssage Regular for 10 s four times (Group 1); four cycles of mechanical cleaning with Merssage Regular for 10 s and Merssage Fine for 10 s (Group 2); four cycles of mechanical cleaning with Merssage Regular for 10 s and Merssage Fine for 30 s (Group 3); and mechanical cleaning with Merssage Fine for 10 s four times (Group 4). A glossmeter was used to measure surface gloss before and after mechanical cleaning, and a contact stylus profilometer was used to measure surface roughness (Ra).

**Results:**

Polishing with prophylactic paste led to a significant reduction in surface gloss and increase in surface roughness among resin composite blocks, whereas the polishing-related change in surface gloss or roughness was smaller in Celtra DUO, a zirconia-reinforced lithium silicate block.

**Conclusions:**

Changes in surface gloss and roughness due to polishing with a prophylactic paste containing large particles were not improved by subsequent polishing with a prophylactic paste containing fine particles.

** Key words:**CAD/CAM, professional dental prophylaxis, prophylactic paste, surface gloss, surface roughness.

## Introduction

Due to recent notable advances in computer-aided design/computer-aided manufacturing (CAD/CAM) technology in dentistry, the application of CAD/CAM technology in coronal restoration is spreading rapidly in Japan (1). Along with the dissemination of the technology, it has become possible to obtain various types of ceramic blocks, such as lithium disilicate blocks, leucite-reinforced blocks, and blocks containing zirconia or alumina, in addition to conventional leucite-based glass ceramic blocks (2). Furthermore, because of the approval of CAD/CAM-fabricated resin composite indirect restorations in premolars for national health insurance coverage in 2014, CAD/CAM technology-based metal-free dentistry is expected to advance quickly, replacing alloys containing gold, silver, and palladium, which have been used widely for molar restorations in Japan.

In addition, there has been growing interest in oral care due to the increasing preference for cleanliness among patients. Many patients now visit dental clinics every 3-6 months for professional dental prophylaxis, also known as professional mechanical tooth cleaning (PMTC), by dental hygienists after the completion of treatment for caries or periodontal disease, crown prosthesis, or a series of other dental treatments. Professional dental prophylaxis effectively removes biofilms, calculi, and even stains from the tooth surface, improving dental aesthetics (3-5). The patient expects the enamel surface to be smooth and shiny, accompanied by an exhilarating sensation in the mouth, due to the action of the prophylactic paste used in the prophylaxis procedure (6,7).

Because of advances in metal-free dentistry, we encounter an increasing number of treatment scenarios where we provide professional care for metal-free restorative materials, such as composite resins and ceramics, in addition to dental enamel and gold-silver-palladium alloys, both of which are treated in the conventional prophylaxis. However, there are many uncertainties about how professional dental prophylaxis affects the surface texture of metal-free restorative materials.

In this study, to clarify the effect on CAD/CAM restorative materials in routine dental practice, professional dental prophylaxis was performed on two types of CAD/CAM composite blocks and two types of CAD/CAM ceramic blocks to compare the prophylaxis-induced changes in surface roughness and gloss. The null hypotheses of this study were that (1) the pre-prophylaxis surface texture does not differ significantly among the four CAD/CAM restorative materials and (2) that professional dental prophylaxis has no significant effect on the surface texture of the restorative materials.

## Material and Methods

-Materials

 Table 1 shows the restorative materials used in this study: Shofu Block HC (Shofu, Kyoto, Japan), and Estelite Block (Tokuyama Dental, Kamisu, Japan), which are CAD/CAM composite blocks; IPS Empress CAD (Ivoclar Vivadent, Schaan, Liechtenstein), and Celtra DUO (DeguDent GmbH, Hanau-Wolfgang, Germany), which are CAD/CAM ceramic blocks. The prophylaxis pastes used in this study were Merssage Regular (Shofu, Kyoto, Japan) and Merssage Fine (Shofu, Kyoto, Japan).

**Table 1:**
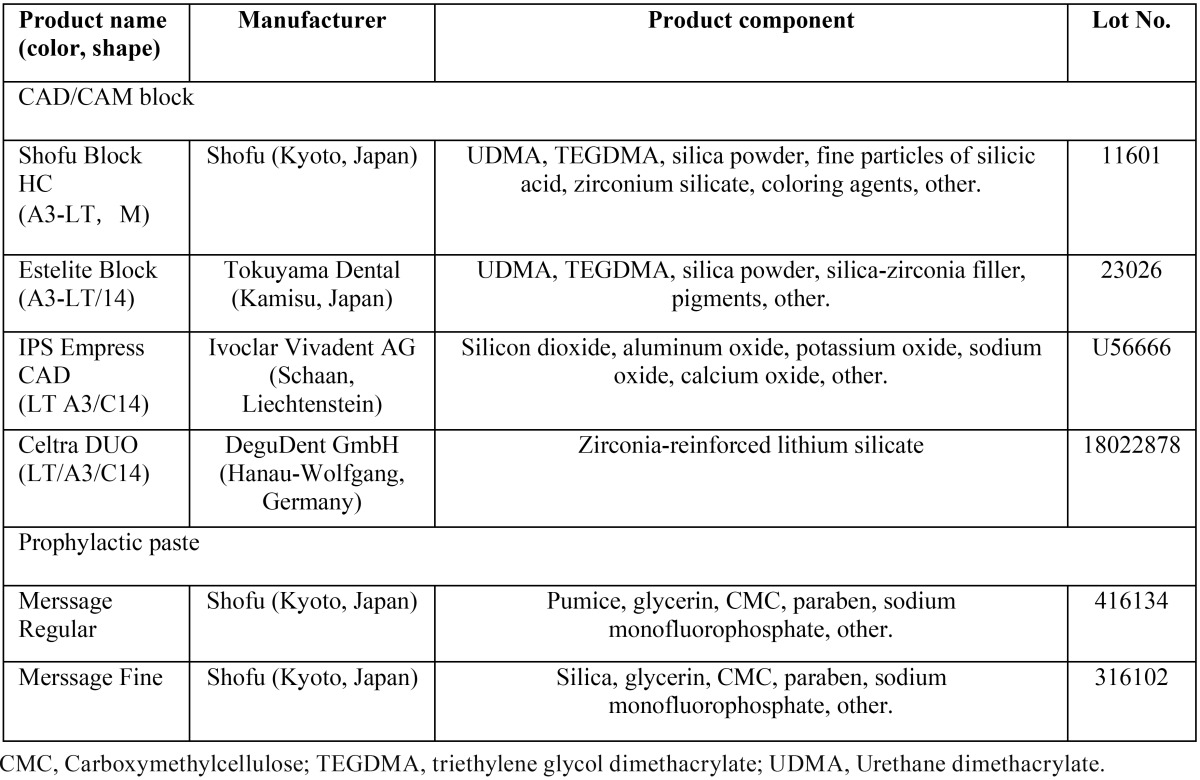
Materials used in this study.

-Sample preparation and experimental groups

Each CAD/CAM material was cut into 20 discs approximately 3 mm thick by using a low-speed diamond saw (Isomet, Buehler, Lake Bluff, IL, USA). Samples were ground using waterproof abrasive papers in the order grit #400, #800, and #1200 and were then polished using alumina suspensions (0.3 and 0.05 nm; Refine Tec, Yokohama, Japan). The samples were randomly divided into four groups (n = 5) for the following prophylaxis procedures.

- Group 1: Merssage Regular for 10 s, four times.

- Group 2: Four cycles of Merssage Regular for 10 s and Merssage Fine for 10 s.

- Group 3: Four cycles of Merssage Regular for 10 s and Merssage Fine for 30 s.

- Group 4: Merssage Fine for 10 s, four times.

-Professional dental prophylaxis

Each sample immobilized on a disposable petri dish was placed on a platform on kitchen scales and prophylaxis paste (0.5 mL) was applied to the center of the sample. Prophylaxis was performed by mounting Merssage Brush No. 2 (Shofu, Kyoto, Japan) on a 16:1 contra-angle slow speed handpiece and operating the brush at 2500 rpm and a load of 200 gf. After every prophylaxis procedure, the sample was washed with water and was air-dried.

-Surface gloss (Gs(60°)) measurement

Measurements were performed on three items before and after professional dental prophylaxis. Surface gloss was measured at a specular angle of 60° by using a precision glossmeter (GD-26, Murakami Color Research Laboratory, Tokyo, Japan) with the light source and detector both set at 60° to the normal. Before measurement, the glossmeter was calibrated to a standard gloss board (Gs(60°) = 92.1%). Measurements were performed at five sites near the center of each sample to calculate the mean Gs value.

-Surface roughness (Ra)

Surface roughness was measured using a surface profilometer (Surfcom 130A, Tokyo Seimitsu, Tokyo, Japan), with a standard cutoff of 0.8 mm, a transverse length of 0.8 mm, and a stylus speed of 0.6 mm/s. By changing angles, measurements were performed at five sites near the center to calculate mean surface roughness (Ra).

-Change in sample weight

Sample weight was measured before and after prophylaxis using a digital analytical balance (HR-202i, A&D Co., Tokyo, Japan), and the change in sample weight was calculated.

-Statistical analysis

Statistical analysis was performed using IBM SPSS Statistics 18 for Windows (IBM, Armonk, NY, USA), with significance set at *p*<0.05. The pre-prophylaxis values of surface gloss and roughness among different restorative materials were compared by one-way analysis of variance, followed by Tukey’s honest significant difference (HSD) test. The data were used to calculate the mean and standard deviation (SD) for each group. In addition, a paired t-test was performed to analyze the differences in surface gloss and roughness and sample weights before and after PMTC.

## Results

-Comparison of baseline surface texture

 Table 2 show the baseline values of surface gloss and roughness measured before prophylaxis. Compared with the other materials, Celtra DUO had a significantly higher surface gloss (*p*<0.05), whereas Shofu block HC had a significantly lower surface gloss and higher surface roughness (*p*<0.05).

**Table 2:**
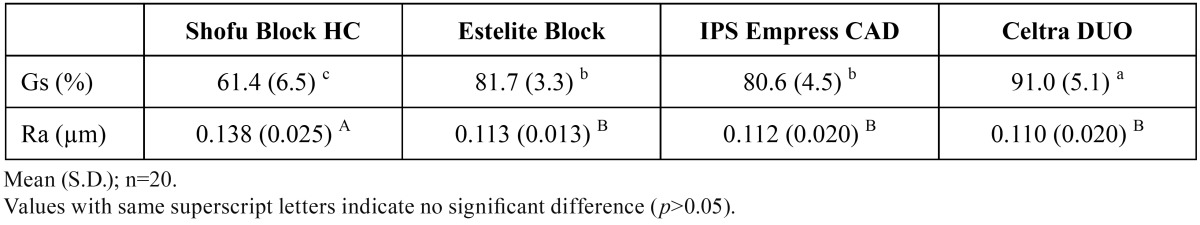
Baseline values of specular gloss at 60° (Gs) and surface roughness (Ra).

-Change in surface gloss

 Table 3 shows the surface gloss values among the four groups measured before and after prophylaxis. After prophylaxis, Shofu block HC and Estelite Block showed a significant reduction in surface gloss in Groups 1-3 (*p*<0.05) but not in Group 4 (*p*>0.05). In all four groups, IPS Empress CAD showed a significant reduction in surface gloss after mechanical cleaning (*p*<0.05), whereas no change in surface gloss was observed in Celtra DUO (*p*>0.05).

**Table 3:**
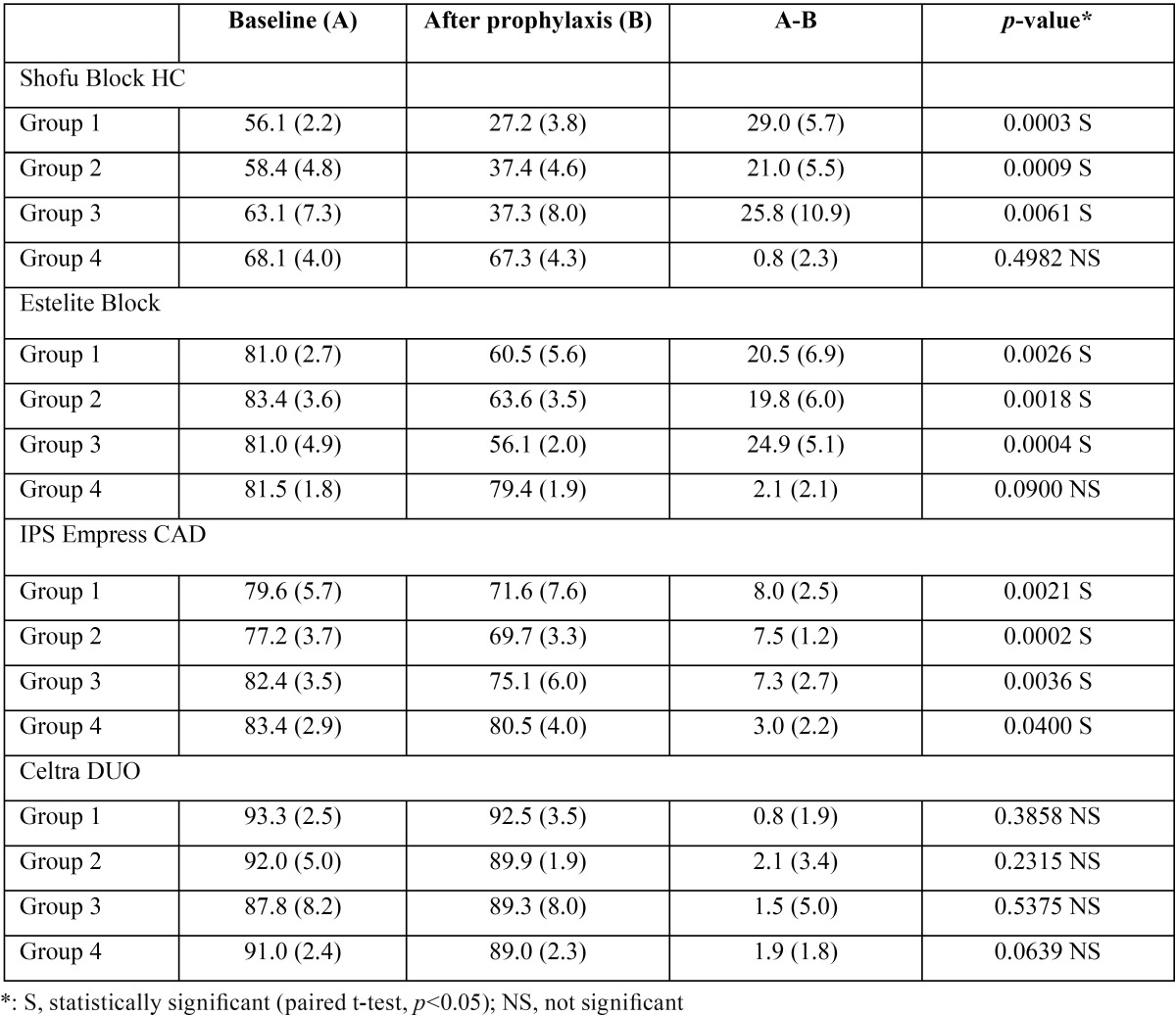
Difference in surface gloss measured before and after prophylaxis (mean (S.D.), %, n=5).

-Change in surface roughness

 Table 4 shows the values of surface roughness among the four groups measured before and after prophylaxis. After mechanical cleaning, the Ra value of Shofu Block HC increased significantly in Groups 1–3 (*p*<0.05) but not in Group 4 (*p*=0.8625). The Ra value of Estelite Block increased significantly in Group 3 (*p*=0.0465) but tended to increase in Groups 1 and 2 (*p*>0.05) or did not change in Group 4 (*p*=0.1006). In all the groups except for Group 2, IPS Empress CAD showed no significant change in surface roughness (*p*>0.05). In addition, Celtra DUO showed no significant change in surface roughness in all four groups (*p*>0.05).

**Table 4:**
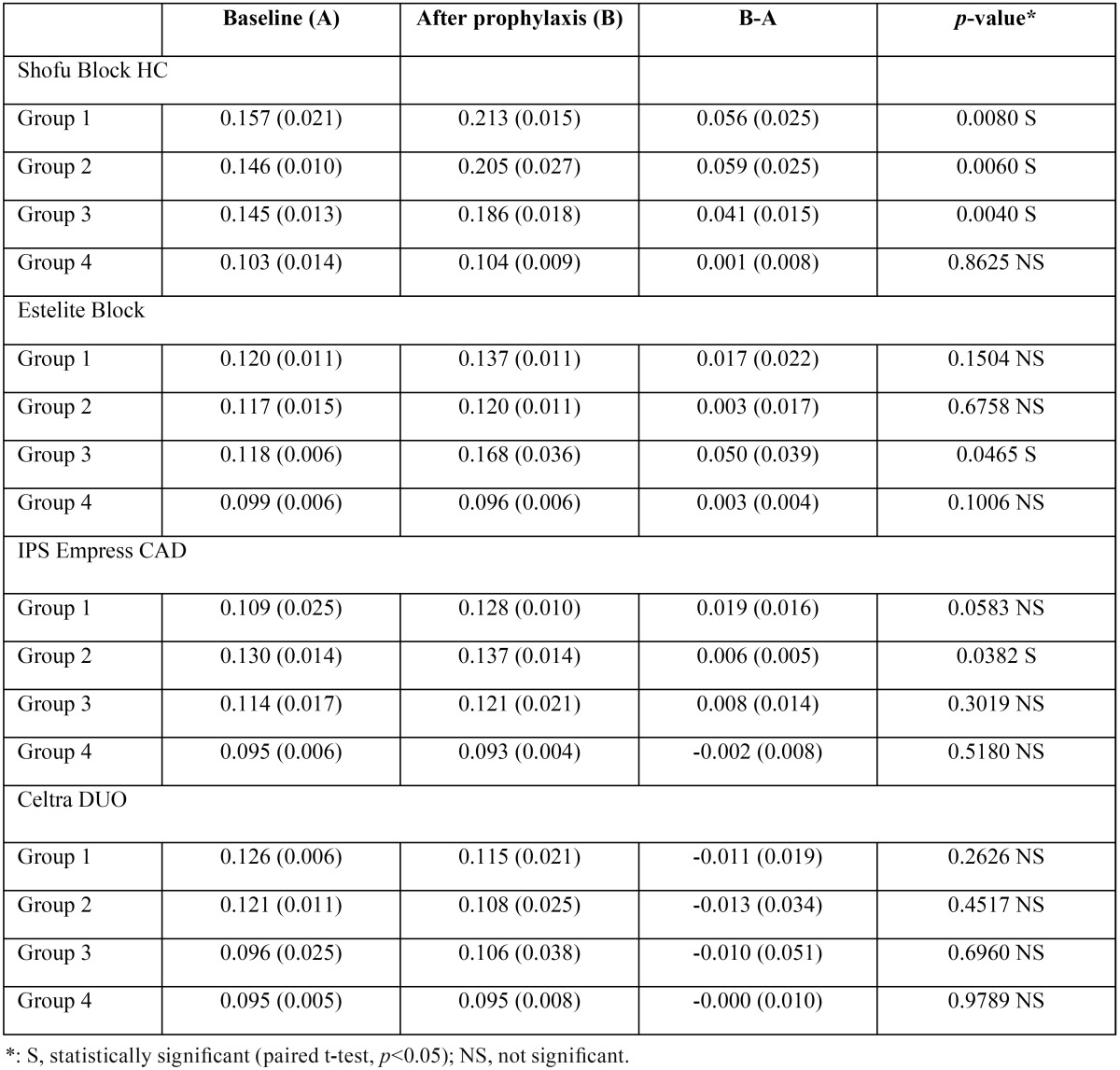
Difference in surface roughness measured before and after prophylaxis (mean (S.D.), µm, n=5).

-Change in sample weight

 Table 5 shows the sample weights measured before and after prophylaxis. After prophylaxis, significant change in weight could not be observed (*p*>0.05).

**Table 5:**
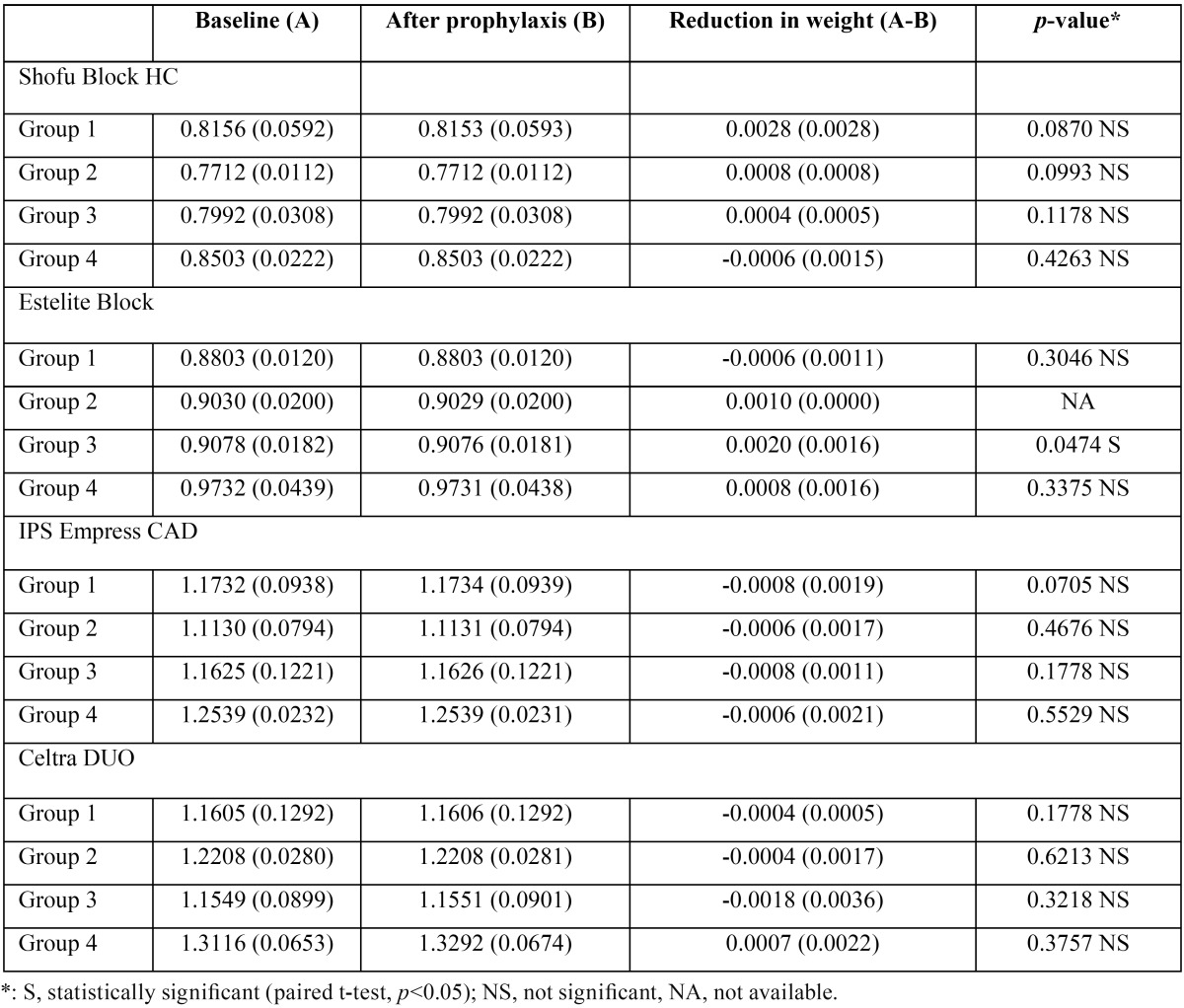
Difference in sample weights measurd before and after prophylaxis (mean (S.D.), g, n=5).

## Discussion

Surface gloss and roughness of four CAD/CAM restorative materials after mirror finish polishing were measured to acquire baseline values. The results showed that Shofu Block HC had a significantly lower surface gloss and higher surface roughness compared with the other three materials, rejecting our first null hypothesis. Our findings were comparable to the findings of previous studies investigating the effect of brush wear (8,9). Both Shofu Block HC and Estelite Block use urethane dimethacrylate (UD-MA) and triethylene glycol dimethacrylate (TEGDMA) as matrix resins and overall contain similar components. However, the content of inorganic fillers is approximately 60% in Shofu Block HC and 75% in Estelite Block. In addition, Shofu Block HC contains a large filler and is regarded as a hybrid composite, whereas the filler in Estelite Block is 150-nm supra-nanoparticles. We previously showed that surface gloss and roughness depend on the mean size and shape of the composite resin fillers (10). Our present results also revealed that the content, size, and shape of filler particles affect the post-polish surface gloss and roughness of CAD/CAM composite blocks.

Despite there being no significant difference in surface roughness, baseline surface gloss was significantly higher in Celtra DUO, which is a zirconia-reinforced lithium silicate, than in IPS Empress CAD, which is classified as a leucite-based glass ceramic and is relatively fragile compared to lithium silicate ceramic (11,12). In a previous study, extracted molars were subjected to root canal formation, filling, and then coronal restoration was performed with different restorative materials. In break strength testing, the fracture strength was clearly lower in the molar repaired with IPS Empress CAD than that repaired with Celtra DUO, showing that material properties vary greatly even among ceramics (13).

Professional dental prophylaxis is performed to remove plaques deposited on the surface of teeth or in the subgingival area to treat or prevent dental caries and periodontal disease (14). As revealed by a long-term survey, professional dental prophylaxis is extremely effective in preventing attachment loss and dental caries (15). However, prophylaxis pastes increase the surface roughness of the enamel, dentin, and restorative materials, and the outcome of PMTC may vary depending on the type of restorative materials and pastes (16,17). Therefore, in this study, we measured the surface gloss and roughness of restorative materials before and after four different prophylaxis procedures to clarify the effect of professional dental prophylaxis on surface texture.

The amount of the paste and the rotational speed and load of the toothbrush were determined prior to performing prophylaxis. In general, prophylaxis procedure is performed at 1000–3000 rpm (17-20). In this study, a 16:1 slow-speed contra-angle handpiece was connected to a dental micromotor that rotates at up to 40,000 rpm, and prophylaxis was performed at 2500 rpm. In addition, considering the effect of force used during prophylaxis procedure on surface gloss and roughness, the force was maintained at 200 gf by placing each sample on a kitchen scale and manually operating the handpiece.

In general, professional dental prophylaxis is performed every 3 months, that is, four times a year, and one side of tooth requires approximately 7–20 s to clean. Based on the information, we simulated how 1 year of prophylaxis affects the surface gloss and roughness of CAD/CAM blocks.

When prophylaxis was performed using Merssage Regular, which contains large particles and has a relative dentin abrasivity (RDA) value of 140–170 (Group 1), a significant decrease in surface gloss and a significant increase in surface roughness were observed in Shofu Block HC, Estelite Block, and IPS Empress CAD, suggesting that using the prophylactic paste produced micro scratches.

In daily clinical practice, professional dental prophylaxis with a paste containing large particles is followed by a prophylactic paste containing small particles to smooth and polish the surface. However, when Merssage Regular was used, even the application of Merssage Fine with an RDA value of 40–50 failed to restore the surface gloss and roughness to the original levels (Group 2). In addition, extended cleaning with Merssage Fine had no significant effect on the outcome (Group 3). These findings suggest that recovery is difficult once the surface of restorative materials is roughened. It is difficult to remove extensive stains and plaques completely using Merssage Fine alone in routine clinical practice. In such cases, the concurrent use of Merssage Regular and Fine is inevitable. In other words, the use of regular pastes, such as Merssage Regular, should be avoided in patients who maintain good oral hygiene and keep plaques under control. To minimize bacterial retention, mean surface roughness (Ra) needs to be ≤ 0.2 µm (21). In this study, the mean Ra value of Shofu Block HC was above 0.2 µm in Groups 1 and 2. This suggests that the use of Shofu Block HC in patients with poor oral hygiene may facilitate plaque buildup.

In this study, PMTC had no adverse effect on the surface texture of Celtra DUO. This means that the second null hypothesis was rejected for Shofu Block HC, Estelite Block, and IPS Empress CAD, but not for Celtra DUO. According to a previous study, Celtra DUO is a strong, stiff, hard CAD/CAM restorative material due to its high flexural strength, elastic modulus, and Vickers hardness values (22). Furthermore, its wear pattern has been reported to be extremely smooth (22,23). We consider that these properties have contributed to the maintenance of high surface gloss and low surface roughness even after prophylaxis in Celtra DUO.

This study investigated the effect of professional dental prophylaxis on the surface gloss and roughness of four types of CAD/CAM indirect restorative materials: Shofu Block HC, Estelite Block, IPS Empress CAD, and Celtra DUO. Our findings were as follows: (1) After mirror finish polishing, baseline surface gloss and roughness values were significantly smaller and larger, respectively, in Shofu Block HC than in the other three materials; and (2) polishing with a prophylactic paste significantly reduced surface gloss and increased surface roughness in composite resin blocks (Shofu Block HC, Estelite Block), whereas relatively small changes in surface texture were observed in Celtra DUO. In addition, polishing with a fine paste failed to improve the surface gloss or roughness altered by a regular paste containing large particles. Due to the development of composite resin materials that are as smooth and shiny as ceramic materials, such as Estelite Block, it is difficult to distinguish composite resin materials from ceramic materials at first glance. Our findings also revealed that the effect of professional dental prophylaxis depends on the basic composition of ceramics. Therefore, preventive measures should be provided only after fully understanding the condition of plaques, teeth, and periodontal tissue, and considering the properties of preexisting restorative materials and prosthetic devices in the oral cavity.
